# The association between dietary inflammation and fatigue in COPD: a mediating role of systemic inflammation and the moderating effect of BMI

**DOI:** 10.3389/fpubh.2026.1850672

**Published:** 2026-07-03

**Authors:** Jiahui Luan, Chang Zhou, Yuan Wang, Siqi Meng, Yingying Wang, Ruohan Zhang, Yusong Cheng, Kunli Wang

**Affiliations:** 1 School of Nursing and Rehabilitation, Shandong University, Jinan, Shandong, China; 2 The Second Hospital of Shandong University, Jinan, Shandong, China

**Keywords:** COPD, dietary inflammation, fatigue, mediation analysis, systemic inflammation

## Abstract

**Objective:**

Fatigue is a common symptom in COPD patients that severely affects quality of life. This study examined whether systemic inflammation (C-reactive protein, CRP) mediates the association between dietary inflammatory potential (Dietary Inflammatory Index, DII) and fatigue, and whether BMI and smoking moderate this pathway.

**Methods:**

A cross-sectional study of 400 stable COPD patients from Jinan, China, was conducted. Fatigue was assessed using the Manchester COPD Fatigue Scale (MCFS), DII was derived from a Food Frequency Questionnaire, and serum CRP was measured by ELISA. Mediation and moderated mediation analyses (PROCESS Models 4 and 58, Bootstrap = 5,000) were performed, adjusting for age, sex, smoking, lung function, comorbidities, and physical activity. Multi-group structural equation modeling examined smoking’s moderating effect.

**Results:**

DII correlated positively with CRP (*r* = 0.194) and fatigue (*r* = 0.397); CRP correlated positively with fatigue (*r* = 0.496) (all *p* < 0.01). CRP partially mediated the DII–fatigue association, accounting for 8.50% of the total effect. BMI moderated this mediation: the indirect effect was stronger at lower BMI (*β* = 0.07, 95% CI: 0.04–0.11) and became non-significant when BMI exceeded 26.71 (*β* = 0.02, 95% CI: 0.00–0.05). Smoking showed no significant moderating effect in formal between-group comparisons.

**Conclusion:**

Higher dietary inflammation is associated with greater fatigue in COPD, partially mediated by CRP, with BMI moderating this pathway. Anti-inflammatory dietary interventions may be more relevant for patients with lower BMI. Longitudinal studies are needed to confirm causality.

## 1 Introduction

COPD is a major global health challenge affecting the lives of millions of people (1), and has a significant impact on global morbidity, mortality, and healthcare costs (2). Among the many symptoms experienced by COPD patients, fatigue is the second most common symptom after dyspnea (3), with an overall prevalence of approximately 60% across different populations and assessment tools (4). Fatigue not only severely limits patients’ daily activities, exacerbates dyspnea, and leads to a poorer prognosis (5), but also causes significant emotional distress (4), and has been identified as an independent predictor of worsening health status and increased mortality (6). Therefore, systematically assessing fatigue status and its influencing factors in COPD patients holds significant clinical importance for intervening in their fatigue.

Dietary inflammation levels can be measured using the Dietary Inflammation Index (DII), which has been shown to correlate with systemic inflammation levels (7). The DII quantifies the pro-inflammatory or anti-inflammatory potential of an individual’s diet by integrating multiple dietary components known to influence inflammatory processes; a higher DII score indicates higher dietary inflammation levels (8). Numerous studies worldwide have reported that a high DII score is significantly associated with elevated levels of key inflammatory markers in the bloodstream. A meta-analysis encompassing data from multiple countries (9) indicates that higher DII scores are associated with a more significant increase in levels of inflammatory factors such as CRP, IL-6, and TNF-*α*. The European EPIC cohort study showed that individuals with higher DII scores had significantly elevated levels of pro-inflammatory cytokines in their circulation (10). Conversely, anti-inflammatory dietary patterns rich in fruits, vegetables, and whole grains are associated with lower levels of systemic inflammation (11). CRP is produced by liver cells in response to pro-inflammatory cytokines and reflects the body’s overall level of inflammation. Because CRP is less influenced by factors such as gender compared to other inflammatory factors like IL-22 and TNF-*α* (12), and because it has a long half-life and high stability, it offers unique advantages in epidemiological studies and is a widely used inflammatory marker (13). Therefore, this study selected CRP as a biomarker of systemic inflammation.

It is worth noting that a consistent association exists between systemic inflammation and fatigue, a relationship that has been validated in both healthy populations and patients with various chronic diseases. In COPD patients, a state of systemic inflammation is considered one of the primary mechanisms underlying the development of fatigue (14). An analysis of inflammatory factors and fatigue-depression indices in the blood of 120 patients with stable COPD demonstrated an association between systemic inflammatory factors and fatigue (14). Multiple studies have similarly shown that various inflammatory markers, including CRP, are positively correlated with the severity of fatigue in patients with moderate-to-severe COPD (15). Furthermore, meta-analyses of both the general population and patients with chronic diseases consistently indicate that higher levels of inflammation are closely associated with more severe fatigue symptoms (16).

Based on this, this study hypothesizes that there is a certain correlation between dietary inflammation levels and the body’s state of fatigue, and that systemic inflammation plays a significant mediating role in this relationship. Existing studies have reported the association between anti-inflammatory dietary patterns, such as the Mediterranean diet, and the body’s fatigue status (17). A 20-year prospective cohort study of COPD patients found that higher DII was positively correlated with increased fatigue and all-cause mortality (18). This suggests a potential pathway linking dietary inflammatory levels, systemic inflammatory status, and fatigue. Although previous studies have separately reported associations between dietary inflammatory potential and fatigue, it remains unclear whether CRP-reflected systemic inflammation mediates the relationship between DII and fatigue in COPD patients. Moreover, whether BMI and smoking status moderate this mediating pathway has not been examined.

In addition to the aforementioned factors, individual characteristics may also alter these pathways. Body Mass Index (BMI), as a key indicator of an individual’s nutritional status, is closely associated with systemic inflammation levels and fatigue severity in COPD patients. At different levels, there are differences in the body’s inflammatory response characteristics (19), respiratory mechanics (20) and metabolic status (21) vary across different BMI levels, which may influence the strength of the pathway through which dietary inflammation affects fatigue via systemic inflammation. Meanwhile, smoking, as the most critical risk factor for COPD, has been shown to be closely associated with elevated systemic inflammation levels (22), and this systemic inflammation may have synergistic or interactive effects with dietary inflammation (23). Therefore, BMI and smoking status in COPD patients may also play a moderating role in this mediating pathway; however, relevant studies in this population are currently lacking.

In summary, this study aims to investigate the association between dietary inflammation levels and fatigue in COPD patients, examine the mediating role of systemic inflammation, and further analyze the moderating effects of BMI and smoking on this mediating pathway, with the goal of providing a theoretical basis for improving fatigue in COPD patients through dietary intervention.

## 2 Materials and methods

### 2.1 Participants

Participants were recruited from multiple community health service centers in Jinan City, Shandong Province, using convenience sampling. The inclusion criteria were as follows: (1) diagnosed with COPD confirmed by post-bronchodilator spirometry showing FEV_1_/FVC < 0.70, and meeting the 2020 Global Initiative for Chronic Obstructive Lung Disease (GOLD) criteria for stable COPD; (2) willing to participate in this study and sign an informed consent form. Exclusion criteria were as follows: (1) patients with concomitant respiratory diseases, including asthma, acute respiratory infections, and COPD exacerbations within the past month; (2) individuals with significant communication difficulties or severe mental or physical illnesses that prevented cooperation with the study; (3) acute infection within the past 4 weeks (24); (4) use of antibiotics or systemic corticosteroids within the past 4 weeks (25); (5) diagnosed autoimmune diseases such as rheumatoid arthritis (26), systemic lupus erythematosus (27), inflammatory bowel disease (28), psoriasis (29), active malignancy (30), or severe cardiovascular events such as acute myocardial infarction (31) and stroke (32) within the past 3 months; (6) other chronic inflammatory conditions not related to COPD (33). A total of 400 COPD patients were recruited for this study. Recruitment was conducted from March to September 2025 across five community health service centers in Jinan City. Of the 612 eligible individuals contacted, 400 agreed to participate (response rate 65.4%). Initially, 418 patients with stable COPD were enrolled. Among them, 12 had missing CRP data (2.9%) and 7 had missing fatigue data (1.7%). Since the proportion of missing key variables was less than 5%, these cases were excluded by listwise deletion, yielding a final analytic sample of 400 patients. No participants were excluded after data collection based on post-hoc criteria. After receiving training, investigators collected participant data, including general demographic information, fatigue assessments, food frequency questionnaires, and other variables that may influence the results, such as physical activity and smoking status (pack-years).

### 2.2 Fatigue

This study used the Manchester COPD Fatigue Scale (MCFS) to assess patients’ fatigue symptoms. This scale was developed in 2009 by AI-shair (34) specifically for patients with stable COPD (GOLD stages 1–4, age ≥40 years). The scale uses 27 items to provide a multidimensional assessment of fatigue experiences in patients with chronic obstructive pulmonary disease. Each item employs a 5-point Likert scale, and the raw total score for each dimension is calculated by summing the scores of all items within that dimension. The MCFS has demonstrated good reliability and validity among Chinese patients with chronic obstructive pulmonary disease; its total score has an internal consistency Cronbach’s alpha of 0.972 and a test–retest reliability of 0.972 (35). In this study, the Cronbach’s alpha for this scale was 0.970.

### 2.3 Dietary patterns

Participants’ dietary intake and DII were assessed using the FFQ. This questionnaire categorizes foods into 14 major groups, encompassing over 150 food items. Its core principle involves estimating total intake of nutrients and food groups by asking participants about the frequency and portion sizes of various foods consumed over a recent period, thereby calculating dietary inflammation levels. This study selected 26 dietary components from the FFQ (36), including energy, protein, carbohydrates, dietary fiber, total fat, saturated fat, monounsaturated fatty acids (MUFAs), polyunsaturated fatty acids (PUFAs), cholesterol, vitamin A, beta-carotene, vitamin B1, vitamin B2, niacin, vitamin B6, total folate, vitamin B12, vitamin C, vitamin D, vitamin E, magnesium, iron, zinc, selenium, caffeine, and alcohol. Each component is compared to the global average intake to calculate a standardized Z-score, which is then doubled and subtracted by 1 to obtain the “food-item-specific DII score.” Summing the DII scores for all dietary components quantifies an individual’s dietary inflammation level (8). Previous studies have reported a median Cronbach’s alpha coefficient of 0.373 for the FFQ across multiple measurements (37). In the present study, the Cronbach’s alpha was 0.423, which is higher than this previously reported median level. Although the Cronbach’s alpha of the FFQ was relatively low in this study, FFQs assess heterogeneous dietary components rather than a single latent construct; therefore, internal consistency is not the primary criterion for evaluating FFQ quality. In addition, In Chinese populations, both the correlation coefficient and ICC of this FFQ were >0.40 (38), indicating its satisfactory reliability in the same cultural and dietary context as our sample.

### 2.4 Covariates and measurements

Other clinical covariates included age, gender, smoking status, FEV_1_% predicted, number of chronic diseases, and physical activity.

For the measurement of FEV_1_% predicted, researchers used a standard-compliant, regularly calibrated spirometer (manufactured by Beijing Maibang Optoelectronic Instrument Co., Ltd., China; model MSA99) operated by rigorously trained researchers. The measured FEV_1_% was used to assess the severity of airflow limitation. Physical activity was measured using the International Physical Activity Questionnaire Short Form (IPAQ). This questionnaire is a retrospective survey tool suitable for individuals aged 16–69 years. It comprises four categories of physical activity (work or school, housework, transportation, and leisure) and six sections (sitting and sleeping) (39). Patients completed the questionnaire after recalling their physical activity over the past 7 days. The IPAQ has good reliability and validity, with a Cronbach’s *α* of 0.73 for the overall questionnaire and 0.744 in this study.

### 2.5 Blood sample collection and measurement

Researchers collected 5 mL of fasting peripheral venous blood from the subjects, placed it into heparin anticoagulant tubes, and centrifuged it at 5,000 r/min for 10 min using a TG16W micro high-speed centrifuge (Hunan Xiangyi Instrument Co., Ltd.). The separated serum was preserved under low-temperature conditions. Inflammatory markers were measured using enzyme-linked immunosorbent assay (ELISA), with kits provided by Wuhan Benlybio Technology Co., Ltd., and the detection instrument was an AMR-100 microplate reader (Hangzhou Allsheng Instruments Co., Ltd.). This study measured the concentration of C-reactive protein (CRP) in the blood of patients with COPD.

### 2.6 Statistical analysis

Data processing and statistical analysis were performed using SPSS 27.0 software. Categorical variables were expressed as frequencies (percentages), and continuous variables were described as mean ± standard deviation. Pearson correlation analysis was used to assess the associations among key variables. The set of covariates in the core model of this study included: age, gender, pack-years of smoking, FEV_1_% of predicted, number of comorbidities, and physical activity level. For the mediation analysis, the PROCESS macro developed by Hayes (Model 4) was used, with the log-transformed inflammatory marker CRP as the mediating variable. With Bootstrap = 5,000, the indirect effect and its 95% confidence interval were calculated to test the mediating effect of CRP between DII and fatigue; the moderating effect of BMI was tested using the PROCESS macro (Model 58). This model assumes that BMI moderates both the forward and backward paths of the mediation process. To more precisely elucidate the moderating pattern of BMI, the Johnson-Neyman method was employed to calculate the region of significance for the moderator BMI, thereby determining the specific range of BMI values at which the effects of DII on CRP and the effects of CRP on fatigue reach statistical significance.

To examine whether smoking status plays a moderating role in the mediation model, a multi-group analysis was conducted. Volunteers were first divided into smoking and non-smoking groups, and then a structural equation model was constructed using AMOS 28 software to test the relationships between DII, CRP, and fatigue separately in each group. Model fit was evaluated using the chi-square-to-degrees-of-freedom ratio (*χ*^2^/df), the Comparative Fit Index (CFI), the Goodness-of-Fit Index (GFI), and the Root Mean Square Error of Approximation (RMSEA). Finally, the Critical Ratios for Differences between Parameters (CRD) method was used to test whether differences in path coefficients between the two groups were statistically significant.

All continuous variables were mean-centered prior to analysis. A bias-corrected Bootstrap method was used to conduct 5,000 repeated samples to estimate confidence intervals for each path coefficient and conditional indirect effect. If the Bootstrap 95% CI did not include 0, the effect was considered statistically significant. All analyses used two-tailed tests with *α* = 0.05.

To address potential common-method bias arising from the collection of all data via online self-administered questionnaires, this study employed Harman’s one-factor test (40) for statistical diagnostics. All measurement items were included in exploratory factor analysis without rotation to examine the variance explained by the first principal component in the unrotated factor solution. The results showed that the first factor explained 18.19% of the total variance, which is far below the critical threshold of 40%. This result indicates that the common method bias in this study is not severe and does not pose a significant threat to the validity of the study’s conclusions.

## 3 Results

### 3.1 Sample characteristics

This study included 400 participants with a mean age of 67.87 ± 10.33 years, of whom 215 (53.75%) were male. The mean BMI was 24.31 ± 4.45 kg/m^2^. The majority were married (83.50%), and 50.25% had a high school education or higher. Regarding clinical characteristics, the mean FEV_1_% predicted was 63.82 ± 19.99%; GOLD stages 1, 2, 3, and 4 accounted for 18.50%, 55.50%, 21.00%, 5.00% of the sample, respectively. Dyspnea assessed by mMRC showed that 91.8% of patients had grades 0–2. Medication for COPD was used by 209 patients (52.25%), and 41 patients (10.3%) required home oxygen therapy. The most prevalent comorbidities were hypertension (30.5%), dyslipidaemia (9.5%), and diabetes (4.0%); regarding comorbidity burden, 129 (32.25%) had no comorbidities, 182 (45.50%) had 1–3, and 89 (22.25%) had more than 3. The mean IPAQ score was 3,501.67 ± 8770.53 MET-min/week. The mean DII, CRP, and fatigue scores were 0.73 ± 1.29, 7.63 ± 4.79 mg/L, and 52.78 ± 26.91, respectively (Table 1).

**Table 1:** Sample characteristics (*n* = 400).

Variable	Mean ± SD or *N* (%)
Demographics	
Age (years)	67.87 ± 10.33
Gender	
Male	215 (53.75%)
Female	185 (46.25%)
BMI (kg/m^2^)	24.31 ± 4.45
Marital status	
Married	334 (83.50%)
Single, widowed, and others	66 (16.50%)
Education	
High school or higher	201 (50.25%)
Illiterate/elementary/middle school	199 (49.75%)
Clinical characteristics	
Disease severity (FEV_1_% predicted)	63.82 ± 19.99
GOLD stage	
GOLD 1 (FEV_1_% ≥ 80%)	74 (18.50%)
GOLD 2 (50% ≤ FEV_1_% < 80%)	222 (55.50%)
GOLD 3 (30% ≤ FEV_1_% < 50%)	84 (21.00%)
GOLD 4 (FEV_1_% < 30%)	20 (5.00%)
Dyspnea level (mMRC grade)	
Grade 0	166 (41.50%)
Grade 1	125 (31.30%)
Grade 2	76 (19.00%)
Grade 3	32 (8.00%)
Grade 4	1 (0.30%)
Medication use	
Yes	209 (52.25%)
No	191 (47.75%)
Home oxygen therapy	
Yes	41 (10.30%)
No	359 (89.80%)
Comorbidities	
Hypertension	122 (30.50%)
Dyslipidaemia	38 (9.50%)
Diabetes	16 (4.00%)
Comorbidity burden (FCI)	
No chronic diseases	129 (32.25%)
1–3 diseases	182 (45.50%)
>3 diseases	89 (22.25%)
Lifestyle factors	
Smoking history (pack-years)	383.16 ± 1506.99
Physical activity (IPAQ, MET-min/week)	3501.67 ± 8770.53
Primary study variables	
DII	0.73 ± 1.29
CRP (mg/L)	7.63 ± 4.79
Fatigue (MCFS)	52.78 ± 26.91

### 3.2 Correlations among major study variables

A Pearson correlation analysis was performed on the primary variables in the study, and the results are shown in Table 2. DII was significantly positively correlated with CRP (*r* = 0.194, *p* < 0.01), DII was significantly positively correlated with fatigue (*r* = 0.397, *p* < 0.01), and CRP was also significantly positively correlated with fatigue (*r* = 0.496, *p* < 0.01). These correlations indicate that as dietary inflammation levels increase, patients’ systemic inflammation levels and fatigue levels both show an upward trend. However, there were no significant correlations between BMI and DII (*r* = −0.001, *p* > 0.05), CRP (*r* = 0.080, *p* > 0.05), or fatigue (*r* = −0.079, *p* > 0.05). Multicollinearity analysis revealed that the VIF values for each variable ranged from 1.007 to 1.046, well below the critical threshold of 5, indicating no multicollinearity issues among the variables. The results of the correlation analysis preliminarily support the research hypothesis that dietary inflammation, systemic inflammation, and fatigue are associated, laying the foundation for subsequent mediation effect tests.

**Table 2:** Correlations among major study variables.

	Variable	DII	CRP	Fatigue	BMI	VIF
1	DII	–				1.040
2	CRP	0.194^**^	–			1.046
3	Fatigue	0.397^**^	0.496^**^	–		–
4	BMI	−0.001	0.080	−0.079	–	1.007

Values are Pearson correlation coefficients. ^**^p < 0.01.

VIF, Variance Inflation Factor; − indicates VIF not calculated for the dependent variable.

### 3.3 Mediating role of CRP

To further explore the underlying mechanisms of DII’s impact on fatigue, this study conducted a mediation analysis. After controlling for a series of demographic and health-related variables, the results are presented in Table 3: The path coefficient from DII to CRP (*a* = 0.181, *p* < 0.001) and the path coefficient from CRP to fatigue (*b* = 0.233, *p* < 0.001) were both significant; The direct effect of DII on fatigue (*c*′ = 0.151, *p* < 0.001) was significant, and the indirect effect (*a* × *b* = 0.042, *p* < 0.001) was also significant. This mediating effect accounted for 8.50% of the total effect (0.496). The above results indicate that CRP plays a significant partial mediating role between DII and fatigue. Among other variables, BMI (*β* = −0.455, *p* < 0.001) and FEV_1_% predicted (*β* = −0.139, *p* = 0.034) were significantly associated with fatigue (see Table 4; Appendix Table 1).

**Table 3:** Mediation role of CRP in the relationship between DII and fatigue.

Path	Model
Direct paths	
DII → CRP (a)	0.181^***^
CRP → fatigue (b)	0.233^***^
DII → fatigue (c′)	0.151^***^
Effect decomposition	
Indirect effect (a × b)	0.042^***^
95% CI	[0.020, 0.068]
Total effect	0.496^***^
Proportion mediated	8.50%

Unstandardized coefficients are reported. Standard errors are in parentheses. The models control for age, sex, smoking history, FEV_1_%, FCI, and activities. CI, confidence interval. ^***^p < 0.001.

**Table 4:** Multiple linear regression model predicting levels of fatigue.

Model	Predictor	*β* (SE)	*t*	*p*
Fatigue	CRP	0.235 (0.021)	11.021	<0.001
	DII	0.147 (0.021)	6.973	<0.001
	BMI	−0.455 (0.133)	−3.415	<0.001
	FEV1%	−0.139 (0.065)	−2.131	0.034

Values are unstandardized regression coefficients (*β*) with standard errors in parentheses. *T*-values and *p*-values are provided for each predictor.

### 3.4 The moderated mediating model

Regression analysis results indicate that DII has a significant positive predictive effect on CRP (*β* = 0.912, *p* < 0.01), and the interaction term between DII and BMI has a significant predictive effect on CRP (*β* = −1.77, *p* < 0.05), suggesting that BMI significantly moderates the relationship between DII and CRP. Further conditional effect analysis revealed that when BMI was at a lower level (the 16th percentile, i.e., 21.47), the positive effect of DII on CRP was stronger (*β* = 0.27, *p* < 0.001); when BMI was at a moderate level (the 50th percentile, i.e., 23.83), the effect was 0.20 (*p* < 0.001); and when BMI was at a high level (the 84th percentile, i.e., 26.71), the effect weakened to 0.11 and was marginally significant (*p* = 0.054) (see Table 1 for details). A Johnson-Neyman analysis further confirmed the moderating pattern of BMI on the forward path: the results showed that when BMI was below 26.71, the positive effect of DII on CRP was significant (*p* < 0.05); when BMI exceeded 26.71, this effect was no longer significant (*p* > 0.05).

In the model examining the relationship between CRP and fatigue, CRP was found to have a significant positive predictive effect on fatigue (*β* = 0.52, *p* < 0.001), and the interaction term between CRP and BMI was also found to have a significant predictive effect on fatigue (*β* = −0.68, *p* < 0.01), indicating that BMI significantly moderates the relationship between CRP and fatigue. The Johnson-Neyman analysis showed that when BMI was low (<21.47 kg/m^2^), the positive effect of CRP on fatigue was stronger (*β* = 0.26, *p* < 0.001); when BMI was moderate (23.83 kg/m^2^), the effect was 0.24 (*p* < 0.001); when BMI was high (>26.71 kg/m^2^), the effect was 0.21 (*p* < 0.001). This indicates that BMI attenuates the promotional effect of CRP on fatigue, but this effect remains significant across all BMI levels.

**Figure 1:**
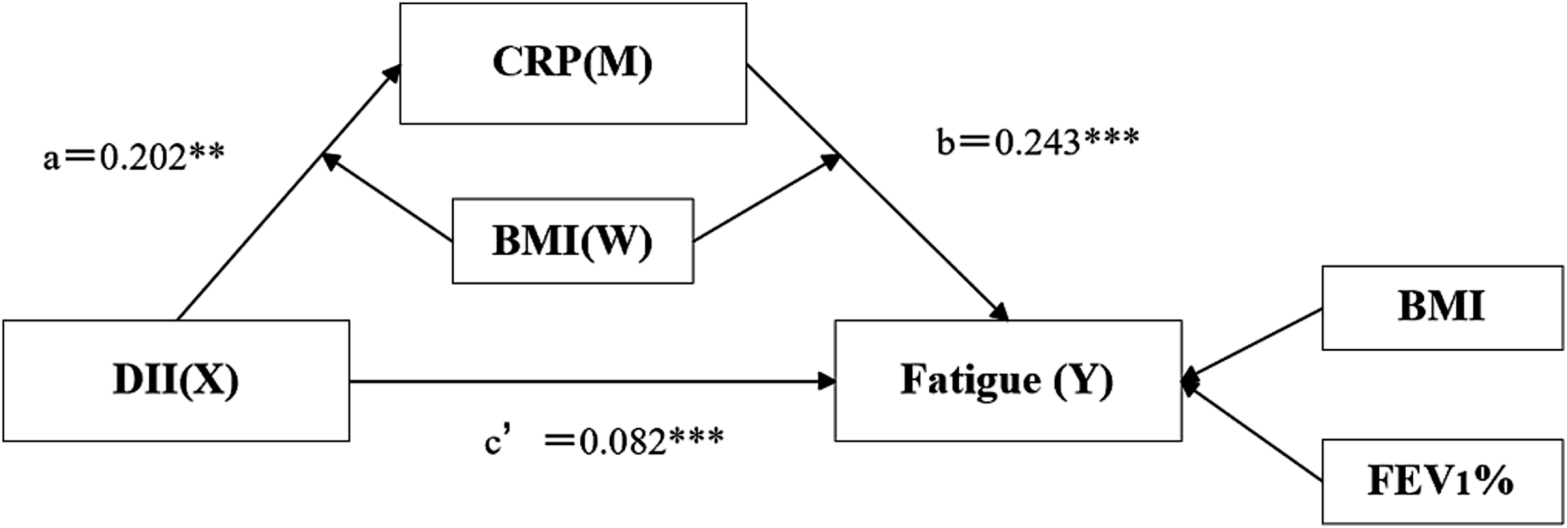
The mediating role of CRP in the relationship between DII and fatigue. Unstandardized path coefficients are shown. See Table 3 for detailed effect decomposition.

Combining the moderating effects of the forward and backward paths, BMI moderated the mediating path between DII, CRP, and fatigue, and the indirect effect gradually weakened as BMI increased (see Tables 5, 6, Figure 1).

**Table 5:** Regression test of the moderated mediating effect.

Variables	CRP				Fatigue			
	*β*	SE	*t*	95% CI	*β*	SE	*t*	95% CI
Constant	0.27	0.25	1.1	[−0.21, 0.76]	0.30	0.13	2.34^*^	[0.05, 0.56]
DII	0.912	0.34	2.72^**^	[0.25, 1.584]	0.14	0.02	6.81^***^	[0.10, 0.18]
BMI	0.973	0.38	2.57^*^	[0.23, 1.72]	−0.05	0.24	−0.22	[−0.41, 0.52]
DII × BMI	−1.77	0.81	−2.19^*^	[−3.36, −0.18]	–	–	–	–
CRP	–	–	–	–	0.52	0.11	4.67^***^	[0.30, 0.73]
BMI × CRP	–	–	–	–	−0.68	0.262	−2.59^**^	[−1.20, −0.16]
*R* ^2^	0.06				0.38			
*F*	2.89^**^				24.29^**^			

^*^p < 0.05, ^**^p < 0.01, ^***^p < 0.001.

**Table 6:** Conditional effects of the mediation model across BMI levels: Johnson–Neyman results.

Path	BMI	Effect	SE	*t*	*p*	LLCI	ULCI
DII → CRP	21.47	0.27	0.06	4.33	<0.001	0.145	0.395
	23.83	0.20	0.05	4.05	<0.001	0.103	0.298
	26.71	0.11	0.06	1.93	0.0542	−0.002	0.230
CRP → fatigue	21.47	0.26	0.02	10.87	<0.001	0.219	0.316
	23.83	0.24	0.02	11.29	<0.001	0.198	0.282
	26.71	0.21	0.02	8.70	<0.001	0.160	0.254

### 3.5 Multi-group analysis by smoking status

To examine whether smoking plays a moderating role in the relationship among DII, CRP, and fatigue, this study grouped patients based on smoking status and conducted a multi-group structural equation modeling analysis. The model fit indices indicated that the model was a well-specified model (*χ*^2^ = 0.000, df = 0, GFI = 1.000, CFI = 1.000), suggesting that the model fits the data perfectly (see Table 7).

**Table 7:** Fit indices for the two multi-group models.

Model	*χ* ^2^	df	*χ*^2^/df	GFI	CFI	RMSEA
Unconstrained model	0	0	–	1	1	–

As shown in Table 8, in the non-smoking group, DII had a significant positive predictive effect on CRP (*β* = 0.197, *p* < 0.001), and CRP had a significant positive predictive effect on fatigue (*β* = 0.429, *p* < 0.001).and the direct effect of DII on fatigue was also significant (*β* = 0.308, *p* < 0.001). In the smoking group, the pathways from CRP to fatigue (*β* = 0.481, *p* < 0.001) and from DII to fatigue (*β* = 0.355, *p* = 0.003) were both significant, but the pathway from DII to CRP did not reach the level of significance (*β* = 0.177, *p* = 0.250).

**Table 8:** Comparison of path coefficients by smoking status.

Path	Group	Unstandardized coefficient (*β*)	SE	CR	*p*	Standardized coefficient (*β*)
DII → CRP	Smoking	0.22	0.191	1.149	0.25	0.177
	Non-smoking	0.187	0.049	3.792	<0.001	0.197
CRP → fatigue	Smoking	0.237	0.06	3.968	<0.001	0.481
	Non-smoking	0.224	0.023	9.735	<0.001	0.429
DII → fatigue	Smoking	0.217	0.074	2.927	0.003	0.355
	Non-smoking	0.153	0.022	6.991	<0.001	

Standardized coefficients (*β*) were derived from the standardized regression weights in the output.

The results of the between-group difference tests are shown in Table 9. The coefficients of the three pathways did not reach statistically significant differences between the smoking and non-smoking groups (the *Z*-values and *p*-values for the DII-to-CRP, CRP-to-fatigue, and DII-to-fatigue pathways were 0.17, 0.865; 0.21, 0.834; 0.85, 0.395). This result indicates that although the predictive effect of DII on CRP in the smoking group did not reach the conventional level of significance, statistical tests found no significant moderating effect of smoking on this path or its overall mediating effect.

**Table 9:** Cross-group difference tests (smokers vs. non-smokers).

Path	Smoking group *B* (SE)	Non-smoking group *B* (SE)	Difference	*Z*-value	*p*-value
DII → CRP	0.220 (0.191)	0.187 (0.049)	0.033	0.17	0.865
CRP → fatigue	0.237 (0.060)	0.224 (0.023)	0.013	0.21	0.834
DII → fatigue	0.217 (0.074)	0.153 (0.022)	0.064	0.85	

*Z* = (*B*_1_ − *B*_2_)/√(SE_1_^2^ + SE_2_^2^), |*Z*| > 1.96 indicates *p* < 0.05.

Appendix 2 shows that in the non-smoking group, the indirect effect of DII on fatigue via CRP was significant (indirect effect = 0.042, 95% CI [0.020, 0.068]); in the smoking group, this indirect effect did not reach statistical significance (indirect effect = 0.052, 95% CI [−0.214, 0.448]). This further supports the observation that smoking status may influence the mediating pathway, but the test for between-group differences did not reach statistical significance.

In summary, smoking status did not exhibit a significant moderating effect in this model. The differences in path coefficients between the two groups did not reach statistical significance, suggesting that the mechanism by which dietary inflammation influences fatigue through systemic inflammation may be similar in both smoking and non-smoking COPD patients. However, the lack of a significant predictive effect of DII on CRP in the smoking group, as well as the phenomenon that the indirect effect was not significant in the smoking group but was significant in the non-smoking group, remains noteworthy. Future studies could employ larger samples or longitudinal designs to further validate the potential moderating role of smoking in this mediating pathway.

## 4 Discussion

In this study, the researchers focused on the mechanisms underlying fatigue in COPD patients. Through mediation analysis and after controlling for covariates, they found that higher dietary inflammatory potential was associated with greater fatigue and higher CRP levels. Mediation analysis suggested that CRP may partly account for the association between DII and fatigue, with the indirect effect accounting for 8.50% of the total effect. This is consistent with Mohammadi et al. (9), Haß et al. (17) and others. Both studies suggest that DII may associate with accelerated increases in systemic CRP levels, and that patients with chronic diseases who consume diets high in pro-inflammatory nutrients are more prone to fatigue, which may be associated with elevated levels of certain blood inflammatory factors (41). This study reaffirms the association between dietary inflammation levels and fatigue, demonstrates that this conclusion holds true in the COPD patient population, and shows that this relationship is mediated, to some extent, by systemic inflammatory processes, thereby providing new evidence regarding the mechanisms underlying fatigue intervention.

A possible explanation for these findings is that a diet with higher inflammatory potential may be associated with increased systemic inflammation, as reflected by CRP levels. Previous studies have suggested that pro-inflammatory dietary patterns may activate inflammatory signaling pathways (42) such as NF-κB (43), is associated with increased oxidative stress, disturb gut microbiota homeostasis (44), and may be linked to insulin resistance (45). These biological processes could provide a plausible background for the observed positive association between DII and CRP in the present study. In turn, elevated systemic inflammation has been linked to fatigue through peripheral mechanisms such as muscle dysfunction (46–48), central nervous system pathways like cytokine-induced sickness behavior (49), and sleep-related disturbances (50). Beyond this indirect pathway through systemic inflammation, a pro-inflammatory diet (51) might also be directly associated with fatigue (52) via other biological routes (53). For example, high DII may influence neurotransmitter metabolism, alter mitochondrial energy production in skeletal muscle (54), or affect neuroendocrine functions such as the hypothalamic–pituitary–adrenal axis (55). These mechanisms could contribute to fatigue independently of CRP-mediated systemic inflammation. Thus, the partial mediating role of CRP observed in our study may represent one potential inflammatory pathway linking dietary inflammatory potential to fatigue in COPD patients, while other direct or indirect pathways may also exist. Despite adjusting for multiple covariates, the relatively weak association between DII and CRP (*r* = 0.194) is not unexpected, as fatigue in COPD is influenced by a wide range of physiological, psychological, and behavioral factors beyond systemic inflammation (56), and CRP levels themselves are determined by genetic, demographic, and lifestyle factors beyond dietary intake (57).

It is worth noting that in this study, CRP mediated only 8.5% of the total association between DII and fatigue, suggesting that the link between DII and fatigue is primarily mediated through pathways other than CRP. A high-DII diet is cross-sectionally associated with disruption of gut microbiota; on the one hand, lipopolysaccharides regulate the onset of fatigue via the gut-brain axis (58), and on the other hand, it is associated with increased fatigue levels by influencing the synthesis of fatigue-related neurotransmitters in the central nervous system (59). At the same time, a high-DII diet is typically accompanied by insufficient intake of nutrients essential for muscle synthesis and metabolism, such as high-quality protein, vitamin A (60), and vitamin E (61), leading to a decrease in muscle mass (62), which is associated with a state of physical fatigue.

In this study, results from a moderated mediation analysis showed that BMI significantly moderated the entire mediation pathway through which DII influences fatigue in COPD patients via CRP. The recommended BMI range for older adults in China is 20.0–26.9 kg/m^2^ (63). In this study, the low BMI (21.47 kg/m^2^) was close to or at the lower limit of the normal range, while the high BMI (26.71 kg/m^2^) had reached the threshold for overweight or obesity. This comparison suggests that the moderating effect of BMI has already shown significant differences within the actual clinical reference range. In the pathway from DII to CRP, the association between DII and CRP was stronger at lower BMI (21.47 kg/m^2^, *β* = 0.27, *p* < 0.001) and weaker to non-significant at higher BMI (26.71 kg/m^2^, *β* = 0.11, *p* = 0.054). Several speculative explanations exist. One possible explanation is that higher BMI may be associated with a state of low-grade inflammation (19), which might attenuate the additional effect of dietary inflammation on CRP levels. Alternatively, compensatory anti-inflammatory mechanisms might buffer the effects of a pro-inflammatory diet in individuals with higher BMI, although this remains speculative (64, 65). Also, higher-BMI individuals may underreport pro-inflammatory food intake, potentially underestimating DII and weakening its association with CRP. In the pathway linking CRP to fatigue, BMI also appeared to moderate this association. The link between CRP and fatigue was stronger at lower BMI (*β* = 0.26, *p* < 0.001) and weaker but still significant at higher BMI (*β* = 0.21, *p* < 0.001). Another possibility is that lower-BMI patients may have lower physiological reserves, making them more susceptible to the effects of systemic inflammation on fatigue (2, 66). In contrast, adipose tissue in higher-BMI individuals may provide an energy buffer during inflammation (67), partially offsetting inflammation-related fatigue. In summary, the indirect effect of DII on fatigue via CRP weakened with increasing BMI and became non-significant at higher BMI. Future studies should incorporate direct measures of body composition, such as fat-free mass index, sarcopenia, and muscle function. This would help to further clarify the moderating role of nutritional status in the inflammation-fatigue pathway. These findings are exploratory, and the explanations remain speculative. The moderating role of BMI should be interpreted cautiously, and longitudinal studies are needed to confirm these pathways. A recent RCT by Toğuç et al. (68) demonstrated that an anti-inflammatory diet significantly reduced both CRP and BMI in obese individuals, supporting the plausibility of our observed pathway and suggesting that the association between dietary inflammation and systemic inflammation may be causally modifiable through dietary intervention, rather than merely correlational.

This study further used smoking status as a grouping variable. However, formal between-group comparisons did not show statistically significant differences in path coefficients. One possibility is that smoking-related chronic inflammation may elevate baseline systemic inflammatory levels, potentially obscuring the additional pro-inflammatory contribution of diet (22). This persistent inflammatory state could reduce the detectable association between DII and CRP. Another possibility is that smokers tend to have less healthy dietary patterns overall (69). Thus, less variability in DII scores among smokers might reduce statistical power to detect the DII and CRP association. Additionally, the generally higher DII scores in smoking COPD patients may create a ceiling effect (70), further limiting the ability to detect incremental associations. However, these interpretations are speculative and need confirmation in larger or longitudinal studies.

This study offers new insights for nutritional interventions aimed at alleviating fatigue in COPD patients. The GOLD 2026 Guidelines (71) state that dietary and nutritional therapy are integral components of comprehensive fatigue management. This study further highlights that dietary composition and dietary inflammatory potential may be relevant targets for future intervention studies aimed at reducing systemic inflammation and fatigue (72). Specifically, it is recommended to increase intake of anti-inflammatory components such as fresh fruits and vegetables, whole grains, and high-quality unsaturated fatty acids, while reducing intake of pro-inflammatory components such as high-sugar, high-fat, and highly processed foods (73). Furthermore, the results of the regression analysis suggest: For patients with low BMI, an anti-inflammatory diet may be a promising target for alleviating fatigue; however, for patients with high BMI, an anti-inflammatory diet alone has limited efficacy, and comprehensive interventions—such as weight loss, improving insulin resistance, and regulating body composition—could be considered as complementary approaches. For smokers, smoking cessation should be the first step to eliminate the pro-inflammatory effects of tobacco smoke on systemic inflammation, followed by anti-inflammatory dietary interventions to more effectively improve fatigue.

This study has the following limitations: First, as a cross-sectional study, it cannot establish a definitive causal relationship between diet, systemic inflammation, and fatigue. Furthermore, the results suggest that diet may influence fatigue through other non-CRP-dependent biological pathways, such as neuroendocrine regulation, gut microbiota metabolism, and mitochondrial function. Future prospective cohort studies and animal models could be used to explore specific mechanisms, thereby more comprehensively revealing the multiple mediating and regulatory mechanisms between diet, inflammation, and fatigue. Second, only serum hs-CRP was measured as an inflammatory marker in our study; other key cytokines such as IL-6 and TNF-*α* were not assessed. Consequently, our mechanistic interpretations are restricted to CRP-mediated pathways, and the broader inflammatory milieu in COPD remains unexplored. Third, the sample for this study was drawn from several community health service centers in Shandong Province, indicating a certain degree of regional limitation. Future research could expand the sampling scope to conduct cross-provincial, multi-center cohort studies and validate this model in populations with different dietary habits, thereby refining the mediating pathways and enhancing the external validity of the conclusions.

## 5 Conclusion

In this cross-sectional study of 400 stable COPD patients, higher Dietary Inflammatory Index (DII) scores were significantly associated with greater fatigue severity. Serum C-reactive protein (CRP) partially mediated this association, accounting for 8.50% of the total effect. The indirect pathway from DII to fatigue via CRP was moderated by BMI: the mediating effect was stronger in patients with lower BMI and became non-significant when BMI exceeded 26.71 kg/m^2^. Although smoking appeared to attenuate the DII–CRP relationship, formal between-group comparisons did not reach statistical significance. These findings suggest that anti-inflammatory dietary interventions might be tailored based on individual BMI, particularly for COPD patients with lower BMI within the normal range. Prospective longitudinal studies and randomized controlled trials are needed to confirm causality and evaluate the long-term efficacy of personalized nutritional strategies.

## Data Availability

The datasets presented in this article are not readily available because some content involves personal privacy. Requests to access the datasets should be directed to Jiahui Luan 202322250062@mail.sdu.edu.cn.
