# Supplementary material for: The association between dietary inflammation and fatigue in COPD: a mediating role of systemic inflammation and the moderating effect of BMI
Source: Front Public Health. 2026 Jul 3;14:1850672. doi: 10.3389/fpubh.2026.1850672 (PMC13375713; doi:10.3389/fpubh.2026.1850672)

**Appendix 1. Multivariate Linear Regression of Fatigue on Age, Number of Chronic Diseases, Physical Activity (IPAQ), and Smoking in COPD Patients**

| Model | Predictor | β(se) | t | p |
| --- | --- | --- | --- | --- |
| Fatigue | Age | 0.021 (0.110) | 0.192 | 0.848 |
|  | Diseases | 0.045 (0.057) | 0.792 | 0.429 |
|  | IPAQ | 0.339 (0.240) | 1.415 | 0.158 |
|  | Smoking | -0.108 (0.087) | -1.237 | 0.217 |

Values are unstandardized regression coefficients (β) with standard errors in parentheses. T-values and p-values are provided for each predictor.

**Appendix 2. Multi-group structural equation model of the mediating pathway in COPD patients stratified by smoking status (smokers vs. non-smokers)**


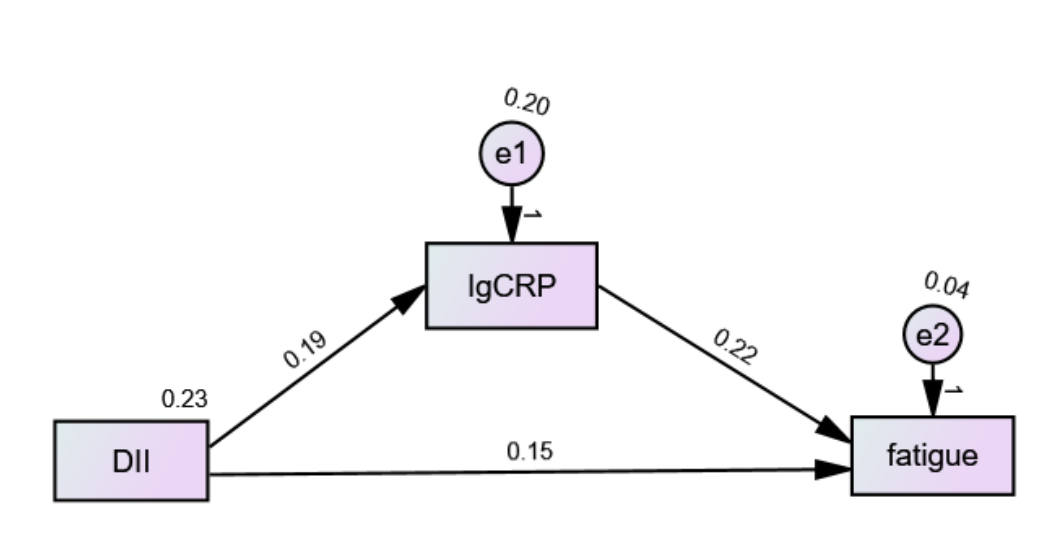

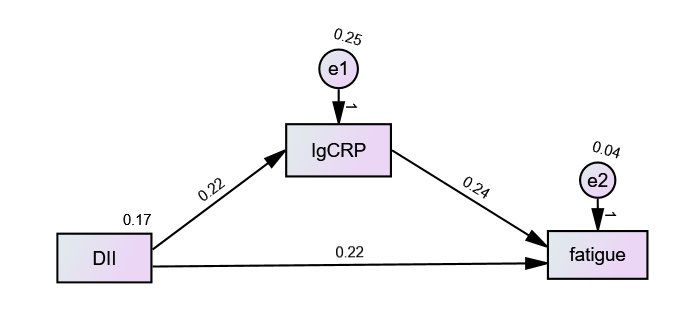

Supplement: Supplementary file 1 [file Data_Sheet_1.DOCX]
